# Absence of stimulus-driven synchronization effects on sensory perception in autism: Evidence for local underconnectivity?

**DOI:** 10.1186/1744-9081-4-19

**Published:** 2008-04-24

**Authors:** Mark Tommerdahl, Vinay Tannan, Jameson K Holden, Grace T Baranek

**Affiliations:** 1 Department of Biomedical Engineering, University of North Carolina at Chapel Hill, Chapel Hill, North Carolina 27599, USA; 2 Department of Allied Health Sciences, University of North Carolina at Chapel Hill, Chapel Hill, North Carolina 27599, USA

## Abstract

**Background:**

A number of neurophysiological characteristics demonstrated in autism share the common theme of under-connectivity in the cerebral cortex. One of the prominent theories of the cause of the dysfunctional connectivity in autism is based on distinct anatomical structures that differ between the autistic and the neurotypical cortex. The functional minicolumn has been identified as occupying a much smaller space in the cortex of people with autism as compared to neurotypical controls, and this aberration in architecture has been proposed to lead to under-connectivity at the local or within-macrocolumn level, which in turn leads to dysfunctional connectivity globally across cortical areas in persons with autism. Numerous reports have indicated reduced synchronization of activity on a large scale in the brains of people with autism. We hypothesized that if the larger-scale aberrant dynamics in autism were due – at least in part – to a widespread propagation of the errors introduced at the level of local connectivity between minicolumns, then aberrations in local functional connectivity should also be detectable in autism.

**Methods:**

Recently, we reported a method for measuring the perceptual changes that are impacted by the presence of synchronized conditioning stimuli on the skin. In this study, the temporal order judgment (TOJ) and temporal discriminative threshold (TDT) of 10 adult autism subjects were assessed both in the absence and presence of synchronized conditioning vibrotactile stimuli.

**Results:**

Our previous report demonstrated that delivering simultaneous and synchronized vibrotactile stimuli to near-adjacent skin sites decreases a subject's ability to determine temporal order by 3 to 4-fold. However, results presented in this report show that subjects with autism do not demonstrate such decreased capacity in temporal order judgment (TOJ) in the presence of synchronized conditioning stimuli, although these same subjects do have TOJ thresholds well above that of controls.

**Conclusion:**

It is speculated that the differences in sensory perceptual capacities in the presence of synchronized conditioning stimuli in autism are due to local under-connectivity in cortex at the minicolumnar organizational level, and that the above-average TOJ thresholds in autism could be attributed to structural differences that have been observed in the frontostrial system of this population.

## Background

Autism is a pervasive developmental disorder that affects many aspects of the central nervous system, including sensory and motor deficits. For example, a number of autism studies have described Parkinson-like motor characteristics and/or postural control problems which could be attributed to deficits of the basal ganglia portion of the frontostriatal system [1,2]. These deficits in sensorimotor control could be derived, in part, from the role that the frontostriatal system plays in an individual's timing perception as well as the coordination that is required between cortical regions during sensorimotor tasks. One relatively simple measure that can be used for the evaluation of a subject's timing perception is ATemporal Order Judgment (TOJ). TOJ is a measure obtained from determining the minimal inter-stimulus interval necessary for a subject to detect the temporal order of two sequentially delivered peripheral stimuli. This metric of timing perception has been shown to be sensitive to lesions to the supplementary motor area (SMA), posterior parietal cortex, and basal ganglia [3,4]. Additionally, these cortical areas have been implicated from significantly elevated TOJ thresholds (worse performance) in subjects with dyslexia [5], dystonia [6-8], and Parkinson's Disease [9]. One goal of our study was to determine if timing perception in subjects with autism would be elevated in a similar fashion.

Although the sensory aspect of an individual's timing perception could play a distinct role in sensorimotor coordination, the lack of larger scale across-cortex integration and coordination of activity across multiple cortical regions has been demonstrated as being characteristic of autism [2,10-12]. Recently, the role of synchronization (or lack of synchronization) in autism has gained a certain degree of prominent attention. Uhlhaas and Singer [13] recently reviewed the experimental evidence that suggests that functional connectivity is reduced in autism, primarily based on fMRI studies [10-12,14-16] that examine the coordinated activity between different areas of the cerebral cortex. Uhlhaas and Singer [13] argued that these data predict that measures of neural synchrony in subjects with autism should be reduced, yet they also pointed out that there are only a small number of studies that actually address such comparisons of synchronization between neurotypical adults and individuals with autism (e.g., [17,18]). From another perspective, there is a large body of evidence that the cerebral cortex of subjects with autism is significantly modified at the minicolumnar level [19]. Casanova and colleagues suggest that this aberrant minicolumnar structure results in the disruption of the inhibitory architecture [20] that is required for normal function in local neural circuitry. They suggest that disruption of functional connectivity at the local minicolumnar level could be responsible for, or strongly correlated with, the dysfunctional connectivity that has been observed across large scale cortical areas, as described in the neural synchrony studies noted above. In this study, we sought to obtain measures addressing the impact that coordinated somatosensory activity in a local cortical region has on subjects with autism.

We recently investigated the impact that stimulus-driven neuronal interactions, evoked by vibrotactile stimuli at dual skin sites which project to adjacent and near-adjacent cortical ensembles, could have on TOJ [21]. In that study, it was reported that delivering weak intensity (low amplitude) but synchronized and periodic vibrotactile stimuli unilaterally to two adjacent digit tips (D2 and D3) significantly and robustly (3–4 fold) degraded a subject's TOJ performance. However, delivery of the same stimulus conditions to bilateral skin sites showed that there was little or no impairment in TOJ performance. One of the conclusions that was drawn from that study was that the stimulus-driven effect of the synchronized conditioning stimuli coordinated the activity of near-adjacent cortical ensembles (such as those representing two fingers used in normal everyday tasks) and consequently, made it more difficult to distinguish one cortical locus from the other as the two stimulus sites were effectively perceptually bound by the stimulus-driven synchronization.

The above-described method that we recently reported involves "forcing" adjacent cortical regions to become synchronized (via stimulus-drive), and then measuring the impact that the cortical-cortical interactions generated by such synchronized activity has on sensory percepts known to be modulated by activity in those same cortical regions. In other words, if the activity in the cortical regions that represent D2 and D3 in somatosensory cortex become synchronized and/or coordinated, it should be more difficult to perform a TOJ task – assuming normal functional connectivity (as was observed in our previous report). If neurologically compromised individuals – such as those with autism – have distinct systemic cortical deficits, and that these deficits extend to local neuronal circuitry connectivity, then the abnormal functional connectivity between adjacent and/or near adjacent cortical ensembles would decrease the effect that stimulus-driven synchronization has on the TOJ task (i.e., performance on the task would not degrade). Therefore, one goal of this study was to determine if synchronized conditioning stimuli would have an impact on TOJ performance in subjects with autism.

## Methods

The subjects were ten males clinically diagnosed with autism (i.e., Autistic Disorder or Asperger Disorder; DSM-IV-TR; [22]), all naïve both to the study design and issue under investigation. Control data used for comparison has been reported in a previous study [21]. Autism subjects were recruited from the University of North Carolina Neurodevelopmental Disorders Research Center Subject Registry. All ten individuals had been previously tested with the Autism Diagnostic Interview – Revised (ADI-R; [23]), the Autism Diagnostic Observation Schedule – Module 4 (ADOS; [24]), as well as the Wechsler Abbreviated Scale of Intelligence (WASI; [25]), and met the diagnostic criteria for autism on the ADI-R. Education levels were as follows: one subject completed the 11^th ^grade, and the remaining nine subjects completed high school. Participants were screened for co-morbid psychiatric diagnoses, peripheral injury, and other conditions that would affect somatosensation. The average ages were 26.1 ± 6.3 yrs for the autism group and 24.2 ± 6.1 yrs for the control group (mean ± stdev). The average IQ scores were as follows: for the autism group, Verbal = 102.3 ± 17.8, Performance = 103.5 ± 18.7, Full-4 = 102.8 ± 17.7; for the control group, Verbal = 112.0 ± 11.0, Performance = 115.3 ± 8.2, Full-4 = 115.6 ± 7.1. No statistical differences were observed between the two groups for either age or IQ. The subjects gave informed consent and were paid $25/hour for their time. The study was performed in accordance with the Declaration of Helsinki, all subjects gave their written informed consent, and procedures were reviewed and approved in advance by an institutional review board.

A two-alternative forced-choice (2AFC) tracking protocol was used to evaluate the temporal order judgment (TOJ) and temporal discriminative threshold (TDT) capacity of each of the ten right hand dominant subjects. The protocol implemented in this study is described in full detail in a previous report [21]. The subject's right arm was rested comfortably on a table surface, and the hand was placed under a portable vibrotactile dual-site stimulator (CM-1; for full description, see [26]). The two probe tips (5 mm diameter each) were positioned at one of two sets of stimulus sites: (1) on the glabrous pads of digits 2 and 3 of the same hand (unilateral condition) or (2) on the glabrous pads of digit 2 of both hands (bilateral condition).

**Figure 1:**
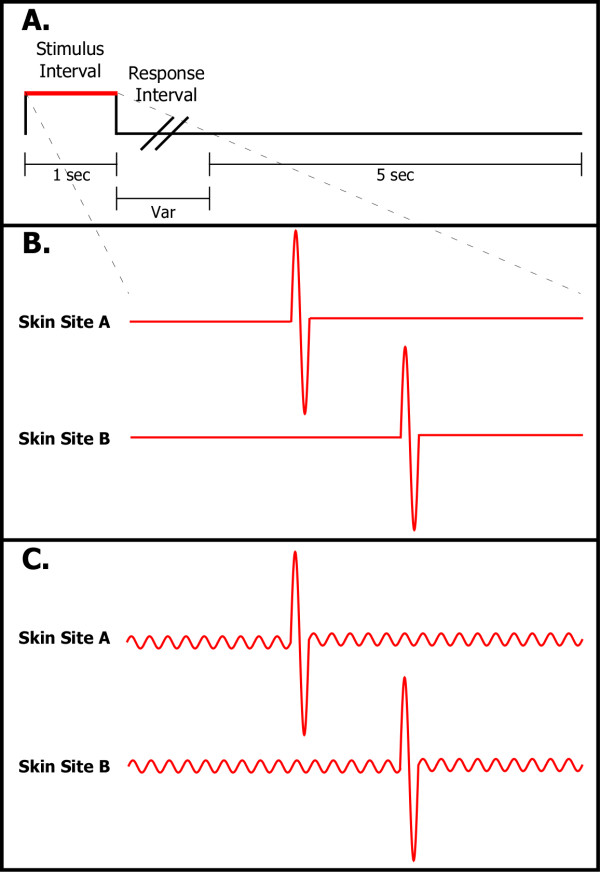
**Protocol details.Panel A: **Two sequential vibrotactile pulses were delivered during the Stimulus Interval, one to each of either skin site A or B. Subject was queried as to which skin site received the first pulse (TOJ) or whether the pulses were synchronous/asynchronous (TDT) during the Response Interval, and this was followed by a 5 sec delay before the onset of the subsequent trial. **Panel B: **Pulse delivery sequence for the TOJ and TDT tasks during each 1 sec Stimulus Interval. Order of delivery (skin site A or B) was randomized on a trial-by-trial basis, and inter-pulse interval was decreased or increased, depending on subject response. **Panel C: **Exemplary 25 Hz conditioning stimulus delivered concurrently with TOJ/TDT task.

At the start of each run, the two probe tips were driven towards the skin sites until each tip registered a force of 0.1 g, as determined by a closed-loop algorithm in the CM-1 stimulator feedback system. The tips were then further indented into the skin by 500 μm to ensure good contact with the skin. The tracking protocol used to obtain individual TOJ and TDT data consisted of 2 separate runs. In one run (20 trials), used for TOJ assessment, two single-cycle vibrotactile test stimuli ("pulses"; 1 mm peak-to-peak amplitude, 25 Hz) delivered to the skin were initially temporally separated by an inter-stimulus interval (ISI) of 150 msec. The locus that received the first of the two pulses was randomly selected on a trial-by-trial basis. The time allocated for stimulus duration was 1 sec (the two 40 ms pulses, separated by the variable ISI, were delivered at the center of this interval), followed by subject response (subject was queried to select the skin site that received the first stimulus) and a 5 sec delay before onset of the next trial (see Panel A of Figure 1). The ISI between the two pulses was modified based on subject response with a 1up/1down algorithm for the first 10 trials and responses for the remaining trials of the run were tracked with a 2up/1down algorithm in which two correct subject responses resulted in a decrement in the ISI. Using a 1up/1down algorithm for the first 10 trials is an efficient way to quickly move the tracking task into a subject's discriminative capacity range without significantly impacting the results [26]. A separate run (also 20 trials) of a similar 2AFC tracking protocol was used for TDT assessment. The TDT protocol differed from the TOJ protocol such that during the stimulus interval, the two pulses were delivered either at the same time or separated temporally by the ISI. Subject response was not dependent on the order in which the two stimuli were delivered, but rather on whether the pulses were felt to be simultaneous or not.

TDT assessment was observed unilaterally and TOJ assessment was observed at both the unilateral and bilateral stimulus sites. In the first condition, there was no concurrent stimulation (control; see Panel B of Figure 1). In the second condition, a 25 Hz concurrent stimulus was delivered (Panel C of Figure 1). During all cases of concurrent stimulus delivery, the concurrent stimulus was delivered for a minimum of 400 msec before the first of the two pulses was delivered and lasted for the entire duration of the allotted interval (1 sec) with the exception of the two 40 ms intervals during which the 1 mm pulses were being delivered. A previous study has reported results obtained from the same described perceptual test with neurotypical adult subjects under multiple conditions of concurrent stimulation [21]. The effects of concurrent stimulation were observed for the unilateral conditions of TDT and TOJ assessment. The order in which the conditions were run was randomized.

## Results

**Figure 2:**
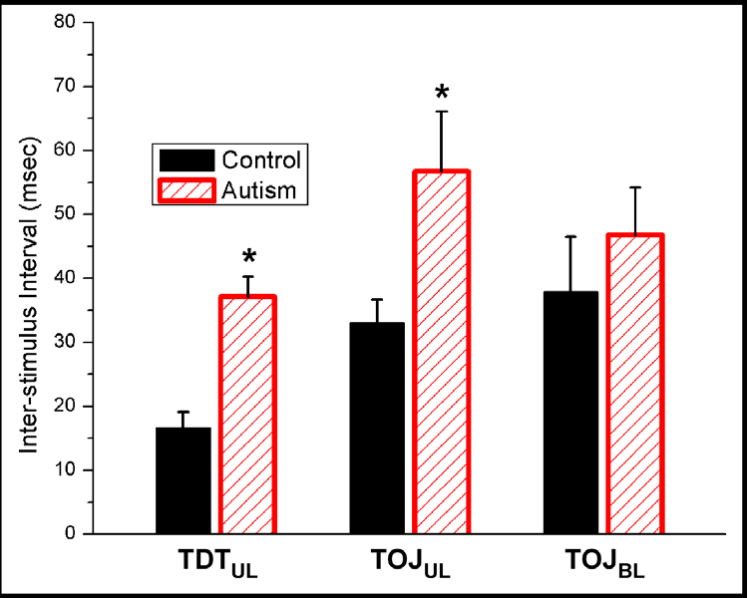
**TDT and TOJ measures obtained at the unilateral D2-D3 and bilateral D2-D2 paired skin sites.** In the unilateral condition, both groups demonstrated lower thresholds for TDT than TOJ (ANOVA; p < 0.01). Furthermore, for each task the control group performed significantly better than the autism group (p < 0.01). In the bilateral TOJ condition, although the control group appeared to perform slightly better at the task, there was no significant difference between the two populations (p = 0.5836).

In order to compare the timing perception of individuals with autism and controls, a two-alternative forced-choice (2AFC) tracking protocol was used to assess discriminative capacities to determine the temporal order of two sequentially delivered tactile stimuli (temporal order judgment; TOJ) and to temporally resolve two sequential stimuli, regardless of order (temporal discrimination threshold; TDT), in individuals with autism. These results were compared with data obtained using an identical protocol from healthy neurotypical controls (control data previously reported in [21]). Figure 2 summarizes the TDT and TOJ measures obtained at the unilateral D2-D3 and bilateral D2-D2 paired skin sites. As was expected, both populations performed significantly better at TDT vs. TOJ (one-way repeated measures ANOVA; p < 0.01). Furthermore, significantly elevated thresholds were observed for the autism group when compared to the controls under both the TDT and TOJ unilateral conditions (p < 0.01). In the bilateral TOJ condition, although the control group appeared to perform slightly better at the task, there was no significant difference between the two populations (p = 0.2836).

**Figure 3:**
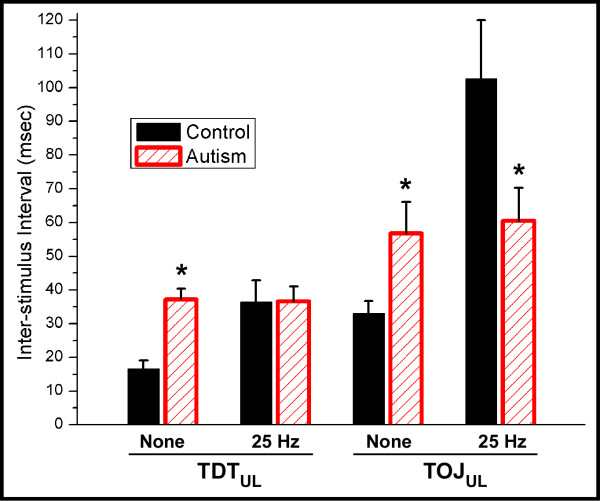
**TDT and TOJ performance metrics obtained under the unilateral conditions in the presence and absence of 25 Hz conditioning stimulation.** Note that for the control group, TDT was significantly elevated (p < 0.01) and TOJ increased 3 to 4-fold (p < 0.01) with 25 Hz conditioning when compared to the condition when no conditioning stimulus was present. In contrast, for both TDT and TOJ measures, conditioning stimulation had no significant impact on the autism group (TDT, p = 0.2561; TOJ, p = 0.4362).

While the TOJ and TDT measures provide an assessment of a subject's timing perception, they do not provide a measure of the impact that the coordinated or synchronized behavior of the adjacent cortical ensembles has on subsequent responses to tactile stimulation. In order to assess whether or not synchronized conditioning stimuli would have an impact on TOJ and TDT, conditioning stimuli were delivered before (a minimum of 400 msec) and concurrently with the TOJ and TDT tasks (see Methods; also [21]). Figure 3 summarizes the TOJ and TDT performance metrics obtained under the unilateral conditions in the presence and absence of 25 Hz conditioning stimulation. Note that for the control group, TDT was significantly elevated (p < 0.01) and TOJ increased 3 to 4-fold (p < 0.01) with 25 Hz conditioning when compared to the respective conditions when no conditioning stimulus was present. In contrast, for both TDT and TOJ measures, conditioning stimulation had no significant impact on the autism group (TDT, p = 0.2561; TOJ, p = 0.4362).

**Figure 4:**
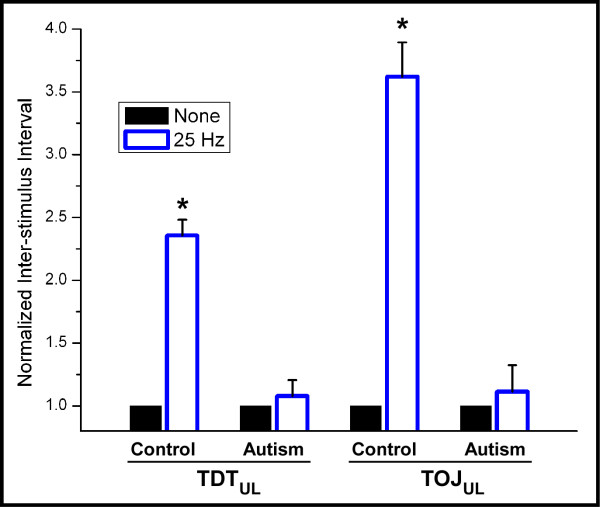
**Data was normalized to the condition during which no conditioning stimulus was present.** The 25 Hz conditioning stimulus significantly impaired TDT by ~240% (p < 0.01) and TOJ by ~360% (p < 0.01) for the control group, whereas the autism group showed no significant change for either measure (p = 0.1986 and p = 0.4329, respectively).

In order to determine whether the differential effects of conditioning observed between the groups were consistent within subjects, the data was normalized to the condition during which no conditioning stimulus was present (shown in Figure 4). The 25 Hz conditioning stimulus significantly impaired TDT by ~240% (p < 0.01) and TOJ by ~360% (p < 0.01) for the control group, whereas the autism group showed no significant change for either measure (p = 0.1986 and p = 0.4329, respectively). The small error bars in the normalized plot confirm that the change in performance due to conditioning was consistent within groups across all the subjects who participated in the study. To summarize, the results suggest two important outcomes: 1) Individuals with autism demonstrated impaired performance on unilateral TDT and TOJ tasks when compared to the control group, and 2) Subjects with autism showed no significant decrement in performance, as do neurotypical controls, on these tasks in the presence of 25 Hz conditioning stimuli.

## Discussion

### Degraded performance of TOJ and TDT in autism

In this study, we made the initial observation that individuals with autism perform significantly worse than neurotypical adults on the TOJ and TDT tasks. The results from our previously reported study [21] demonstrated that neurotypical subjects had TOJ thresholds that were in the range between 30 and 40 msec for both the unilateral (33 ± 4 msec) and bilateral (38 ± 8 msec) conditions. TDT thresholds were found to be around 20 msec for the unilateral (17 ± 3 msec) and bilateral (24 ± 4 msec) conditions, and both measures were well within the range of previously reported values (e.g., [6-8,27-29]). However, the thresholds obtained from subjects with autism were elevated for both unilateral TOJ (57 ± 9 msec) and TDT (37 ± 3 msec), indicating that timing perception in individuals with autism is below normal. Bilateral TOJ measures obtained from subjects with autism and controls were not significantly different, although controls appeared to have lower thresholds (38 ± 8 msec vs. 47 ± 7 msec). Interestingly, within the autism group, performance of the TOJ task in the bilateral condition appears to be slightly better than the unilateral condition, though again – not significantly different.

### Degradation in TOJ could be accounted for by abnormalities in the frontostrial system

Timing perception – as measured by TOJ and TDT – is most often accounted for by the frontostriatal system largely as a result of these timing measures being sensitive to lesions to the supplementary motor area (SMA), posterior parietal cortex, and basal ganglia [3,4], and also because of the fact that above-average TOJ thresholds occur in subjects with known damage to these same cortical areas (dyslexia [5], dystonia [6-8], and Parkinson's disease [9]). In subjects with autism, a number of structures, particularly in the frontostriatal system, have been reported to be compromised and could be responsible for the above average TDT and TOJ thresholds (or below average timing perception) demonstrated in our study. Specifically, several studies implicate the basal ganglia, or disproportionate changes in basal ganglia volume, in autism [30-36]. Langen et al [30] demonstrated an enlargement in the caudate nucleus volume that was disproportionate to increases in total brain volume in subjects with autism. These findings were consistent with the work of others who found increases in basal ganglia volume in individuals with autism [31-33]. Additionally, differences in thalamus volume [37,38], and impaired white matter connectivity in the frontal lobe [39] also implicate the frontostriatal system in the etiology of autism and are consistent with the impairments observed in timing perception in individuals with autism.

### TOJ degrades with synchronized conditioning stimuli – but not in autism

Results from our previous report ([21]; also see Figure 3) showed that when 25 Hz synchronized conditioning stimuli were delivered concurrently at two pairs of unilateral test sites (D2 and D3), neurotypical adult subjects demonstrated a marked decrease in their ability to discriminate temporal order. The results of that study gave support to the theory that synchronization of cortical ensembles in SI could significantly impact the topography of temporal perception. In other words, co-activation of adjacent and/or near-adjacent cortical assemblies makes it more difficult for a subject to perceptually differentiate between the regions of skin that are receiving identical stimulation. However, in the present study, it was observed that there was no degradation in performance in the individuals with autism on the TOJ task in the presence of the same synchronized dual-site conditioning stimuli, and the topography of temporal perception was not impacted. While all control subjects in the previous study tested demonstrated a decreased ability in TOJ in the presence of synchronized conditioning stimuli delivered to unilateral stimulus sites, none of the autism subjects showed a significant alteration in TOJ with the same synchronized conditioning stimuli. The conclusion from the previous study was that the degradation in TOJ observed in controls in the unilateral stimulus condition resulted from the synchronization of activity in adjacent or near-adjacent cortical ensembles which led to those cortical ensembles becoming functionally connected or bound [21]. In the autism subjects, this stimulus-driven synchronization did not lead to degradation of TOJ, suggesting that the engaged cortical ensembles – though in near proximity topographically – are not functionally connected or do not bind. The lack of local functional connectivity between these two topographically proximal regions could be responsible for the absence of perceptual binding that normally occurs with synchronized conditioning stimuli. Recognition of two independent, but identical stimuli, as a single stimulus, could be an indicator of integration on the local cortical level that is important for coordination of sensory input that might play an important role in normal sensorimotor function. However, if individuals with autism do not experience such perceptual binding – or coordination of sensory input – then this "dysfunctional" connectivity could explain a number of enhanced feature extracting capabilities that are often associated with autism [40].

### Changes in local cortical circuitry could lead to changes in under-connectivity and synchronization

The degradation in TOJ performance with stimulus-driven synchronization that is observed in control subjects (but absent in individuals with autism) is presumed to reflect the responses of near-proximal cortical ensembles in primary somatosensory cortex evoked by dual-site skin stimulation. If this presumption is correct, the findings obtained in the present study raise the possibility that local cortico-cortical functional connectivity in subjects with autism may be substantially abnormal. While a number of findings have demonstrated that long-range functional connectivity in subjects with autism is very different from that present in the general population (for example, see [41,42]), it is unlikely that the different observations obtained from individuals with autism and control subjects reported in this study are attributable to differences in long-range cortico-cortical connections. Rather, the measures of the impact that synchronization of topographically proximal cortical ensembles described in this paper appear to reflect the deficit in short-range parietal corticocortical connectivity identified in subjects with autism by Casanova and colleagues [43]. Casanova and colleagues have reported minicolumnar reduction in a number of areas in the parietal cortex, primarily in the peripheral neuropil space surrounding the minicolumn structures [19]. The peripheral neuropil space, being the area that provides the "strong vertical flow of inhibition" described by Mountcastle [44], is the region populated by (inhibitory) double bouquet cells. If the lack of degradation of TOJ in the presence of stimulus-driven synchronization is, in fact, an indicator of altered local circuitry in autism, then it would fit well with the minicolumnar hypothesis of autism that has been put forth by Casanova and colleagues. Such changes in connectivity could lead to the imbalance in excitation and inhibition that others have predicted underlies the neocortical hyperexcitability and unstable activity in cortical networks characteristic of autism [41,45].

### Long range vs. short range functional connectivity

The primary significance of this study is that the lack of perceptual degradation that does not result from stimulus-driven synchronization in autism demonstrates that there is an element of under-connectivity between cortical ensembles at the local regional level. A number of reports have focused on long range functional connectivity – or synchronization between cortical regions across large territories of cortex. These studies that propose long-range cortical under-connectivity in autism predict that there are lower than normal regions of activation that result from a reduction in the number of long range interactions between these areas [14,18,46,47] and that a principle deficit in autism is the coordination of the activity of cortical ensembles across the entire cortex [46]. While the majority of these synchronization studies have been done with fMRI and PET (e.g., [14,46,47]) – which only provides an indirect measure of the dysfunctional neuronal synchrony [48] – a few studies have employed methods, such as MEG, that allow for higher temporal resolution. MEG and EEG studies have found that gamma oscillations, which are considered to be important in the process of coordinating cortical activity, to be below normal in subjects with autism [18,49].

It could be argued that the results from this study have little to do with the synchronization effects that are often considered in functional neuroimaging studies. In those studies, multiple cortical areas become activated in response to a single simple task and the activity of those areas is determined to be highly correlated (or synchronized). However, individuals with autism typically do not exhibit the same degree of connectivity or synchronization as control subjects, and it is from this evidence that a number of under-connectivity theories initially arose [50]. In this study, the perceptual impact that synchronizing adjacent or near-adjacent cortical ensembles was evaluated in order to ascertain functional connectivity at the local cortical level. Proposals of over-connectivity at this local cortical level in autism have been put forth, but much of the evidence for local cortical over-connectivity is anecdotal. Belmonte and colleagues suggested the co-morbidity with epilepsy that is highly prevalent in autism is evidence for over-connectivity [51]. Other reports have suggested over-connectivity or over-processing at the local level, principally because some individuals with autism exhibit hyper-responsive sensory symptoms and/or have enhanced feature processing skills [40,52]. We suggest that perhaps over-connectivity is the incorrect term, and also suggest that our findings strongly support the anatomical findings of Casanova and colleagues. That is, many of the differences – such as those described above – could be accounted for by a higher density of minicolumns and a reduced neuropil surrounding those minicolumns. Although the increase in minicolumn density could account for some enhancements in perception, the reduction in neuropil surrounding adjacent minicolumns would lead to some below-normal perceptual metrics. For example, spatial localization of a stimulus on the skin is much better in individuals with autism than in controls [53], and this could be due to the increased resolution afforded by the higher density of minicolumns. However, because of the lack of GABA-mediated inhibition between those minicolumns, adaptation of the stimulus delivered to the skin – which normally (in healthy adults) results in a nearly 2-fold improvement in spatial localization performance – does not lead to an improvement in spatial localization in autism subjects [53]. Additionally, a degraded adaptive response in autism was recently demonstrated in autism in an amplitude discrimination task, and it was concluded that a generalized GABA deficiency could also account for this behavior [54]. The lack of or reduction of normal inhibitory connectivity between minicolumns in the cortex could, as Casanova has suggested, be responsible for the decrease in larger scale connectivity observed in autism. In other words, if the minicolumn is considered the smallest functional unit of the cortex – which significant neurophysiological evidence suggests [20,44,55-60] – then it stands to reason that the functional connectivity between macrocolumns (which are made up of minicolumns) and aggregates of macrocolumns would lead to a deficiency in larger scale cortical-cortical interactions. We view this idea as consistent with the data presented in this paper that differentiates the impact that synchronizing stimuli have on the perceptual metrics of individuals with autism and control subjects. The method we have developed for detecting the influence of stimulus-driven synchronization, or in the case of autism – the absence of the influence of stimulus-driven synchronization, could be argued to be influenced predominantly by cortical structures at the macrocolumnar, and perhaps the minicolumnar, level. Assessment of the influence of such stimuli on local cortical ensembles is currently being more directly addressed.

One obvious shortcoming of this study that will be addressed in subsequent reports is the question of the relationship of the variability of the measures within the autism subject group and how well this variability correlates with clinical assessments. For example, is the impact of synchronization on TOJ reduced with a decrease in ADOS measures of the subject population? Although the distributions of our current subject populations are essentially non-overlapping in terms of influence of stimulus-driven synchronization (note comparison of normalized conditions in Figure 4), it will be interesting to see if a pattern of stimulus-driven synchronization effectiveness emerges with a larger sample size. Other future studies could include subject populations with abnormalities in somatotopic cortical organization such as those with dystonia, which are known to have disordered digit topographies [61], with the implication that predictions about digit topography could be made from the differential outcomes in TOJ with and without concurrent stimulation. Thus, the metric could be used to complement already existing technologies that are capable of resolving digit topography at high resolution (e.g., [62]) as well as technologies that have examined and detected differences in event-related synchronization/desynchronization in disorders such as Parkinson's, dystonia, physiological aging, degenerative dementia, and obsessive-compulsive disorder (for review, see [63]).

## Conclusion

In our previous report, we concluded that our results suggested that in neurotypical adult subjects – in which functional connectivity between adjacent and/or near-adjacent cortical columns is intact or not impaired – the TOJ measure would be significantly impacted in the presence of a synchronizing stimulus that simultaneously engages paired cortical ensembles. In the case of autism, the results show that the same TOJ measure is not impacted by such synchronizing stimuli, suggesting what a number of reports of cortical microarchitecture have previously suggested in autism: a disruption of local functional connectivity. This seemingly robust measure, which can be obtained relatively quickly and non-invasively, could prove useful in future research assessing the efficacy of treatments for persons not only with autism, but for members of subject populations with other abnormalities in cortical organization.

## Competing interests

The authors certify that the information listed above is complete to the best of our original research. The authors declare that they have no competing interests.

## Authors' contributions

MT and VT participated in the design and conduct of the experiments and the drafting of the manuscript. JKH participated in the conduct of the experiments. GTB participated in the design of the experiments and the draft of the manuscript. All authors read and approved the final manuscript.

## References

[B1] Rinehart NJ, Tonge BJ, Bradshaw JL, Iansek R, Enticott PG, McGinley J (2006). Gait function in high-functioning autism and Asperger's disorder : evidence for basal-ganglia and cerebellar involvement?. European child & adolescent psychiatry.

[B2] Takarae Y, Minshew NJ, Luna B, Sweeney JA (2007). Atypical involvement of frontostriatal systems during sensorimotor control in autism. Psychiatry research.

[B3] Lacruz F, Artieda J, Pastor MA, Obeso JA (1991). The anatomical basis of somaesthetic temporal discrimination in humans. Journal of neurology, neurosurgery, and psychiatry.

[B4] Pastor MA, Day BL, Macaluso E, Friston KJ, Frackowiak RS (2004). The functional neuroanatomy of temporal discrimination. J Neurosci.

[B5] Laasonen M, Tomma-Halme J, Lahti-Nuuttila P, Service E, Virsu V (2000). Rate of information segregation in developmentally dyslexic children. Brain Lang.

[B6] Sanger TD, Tarsy D, Pascual-Leone A (2001). Abnormalities of spatial and temporal sensory discrimination in writer's cramp. Mov Disord.

[B7] Tinazzi M, Frasson E, Bertolasi L, Fiaschi A, Aglioti S (1999). Temporal discrimination of somesthetic stimuli is impaired in dystonic patients. Neuroreport.

[B8] Tinazzi M, Fiaschi A, Frasson E, Fiorio M, Cortese F, Aglioti SM (2002). Deficits of temporal discrimination in dystonia are independent from the spatial distance between the loci of tactile stimulation. Mov Disord.

[B9] Artieda J, Pastor MA, Lacruz F, Obeso JA (1992). Temporal discrimination is abnormal in Parkinson's disease. Brain.

[B10] Kana RK, Keller TA, Cherkassky VL, Minshew NJ, Just MA (2006). Sentence comprehension in autism: thinking in pictures with decreased functional connectivity. Brain.

[B11] Just MA, Cherkassky VL, Keller TA, Kana RK, Minshew NJ (2007). Functional and anatomical cortical underconnectivity in autism: evidence from an FMRI study of an executive function task and corpus callosum morphometry. Cereb Cortex.

[B12] Koshino H, Carpenter PA, Minshew NJ, Cherkassky VL, Keller TA, Just MA (2005). Functional connectivity in an fMRI working memory task in high-functioning autism. NeuroImage.

[B13] Uhlhaas PJ, Singer W (2006). Neural synchrony in brain disorders: relevance for cognitive dysfunctions and pathophysiology. Neuron.

[B14] Castelli F, Frith C, Happe F, Frith U (2002). Autism, Asperger syndrome and brain mechanisms for the attribution of mental states to animated shapes. Brain.

[B15] Villalobos ME, Mizuno A, Dahl BC, Kemmotsu N, Muller RA (2005). Reduced functional connectivity between V1 and inferior frontal cortex associated with visuomotor performance in autism. NeuroImage.

[B16] Cherkassky VL, Kana RK, Keller TA, Just MA (2006). Functional connectivity in a baseline resting-state network in autism. Neuroreport.

[B17] Grice SJ, Spratling MW, Karmiloff-Smith A, Halit H, Csibra G, de Haan M, Johnson MH (2001). Disordered visual processing and oscillatory brain activity in autism and Williams syndrome. Neuroreport.

[B18] Wilson TW, Rojas DC, Reite ML, Teale PD, Rogers SJ (2007). Children and adolescents with autism exhibit reduced MEG steady-state gamma responses. Biological psychiatry.

[B19] Casanova MF, Buxhoeveden DP, Switala AE, Roy E (2002). Minicolumnar pathology in autism. Neurology.

[B20] Casanova MF, Buxhoeveden D, Gomez J (2003). Disruption in the inhibitory architecture of the cell minicolumn: implications for autisim. Neuroscientist.

[B21] Tommerdahl M, Tannan V, Zachek M, Holden JK, Favorov OV (2007). Effects of stimulus-driven synchronization on sensory perception. Behav Brain Funct.

[B22] APA (2000). Diagnostic and statistical manual of mental disorders, 4th edition, text revision.

[B23] LeCouteur A, Lord C, Rutter M (2003). Autism Diagnostic Interview-Revised (ADI-R).

[B24] Lord C, Rutter M, Dilavore P, Risi S (1999). The Autism Diagnostic Observation Schedule (ADOS).

[B25] Wechsler D (1999). WASI Manual.

[B26] Tannan V, Dennis RG, Zhang Z, Tommerdahl M (2007). A portable tactile sensory diagnostic device. J Neurosci Methods.

[B27] Hirsh IJ, Sherrick CE (1961). Perceived order in different sense modalities. J Exp Psychol.

[B28] Fiorio M, Tinazzi M, Bertolasi L, Aglioti SM (2003). Temporal processing of visuotactile and tactile stimuli in writer's cramp. Ann Neurol.

[B29] Craig JC, Xu BH (1990). Temporal order and tactile patterns. Perception & psychophysics.

[B30] Langen M, Durston S, Staal WG, Palmen SJ, van Engeland H (2007). Caudate nucleus is enlarged in high-functioning medication-naive subjects with autism. Biological psychiatry.

[B31] Herbert MR, Ziegler DA, Deutsch CK, O'Brien LM, Lange N, Bakardjiev A, Hodgson J, Adrien KT, Steele S, Makris N, Kennedy D, Harris GJ, Caviness VS (2003). Dissociations of cerebral cortex, subcortical and cerebral white matter volumes in autistic boys. Brain.

[B32] Hollander E, Anagnostou E, Chaplin W, Esposito K, Haznedar MM, Licalzi E, Wasserman S, Soorya L, Buchsbaum M (2005). Striatal volume on magnetic resonance imaging and repetitive behaviors in autism. Biological psychiatry.

[B33] Sears LL, Vest C, Mohamed S, Bailey J, Ranson BJ, Piven J (1999). An MRI study of the basal ganglia in autism. Progress in neuro-psychopharmacology & biological psychiatry.

[B34] Voelbel GT, Bates ME, Buckman JF, Pandina G, Hendren RL (2006). Caudate nucleus volume and cognitive performance: Are they related in childhood psychopathology?. Biological psychiatry.

[B35] Haist F, Adamo M, Westerfield M, Courchesne E, Townsend J (2005). The functional neuroanatomy of spatial attention in autism spectrum disorder. Developmental neuropsychology.

[B36] Rojas DC, Peterson E, Winterrowd E, Reite ML, Rogers SJ, Tregellas JR (2006). Regional gray matter volumetric changes in autism associated with social and repetitive behavior symptoms. BMC psychiatry.

[B37] Hardan AY, Girgis RR, Adams J, Gilbert AR, Keshavan MS, Minshew NJ (2006). Abnormal brain size effect on the thalamus in autism. Psychiatry research.

[B38] Hardan AY, Girgis RR, Adams J, Gilbert AR, Melhem NM, Keshavan MS, Minshew NJ (2008). Brief Report: Abnormal Association Between the Thalamus and Brain Size in Asperger's Disorder. J Autism Dev Disord.

[B39] Lee JE, Bigler ED, Alexander AL, Lazar M, DuBray MB, Chung MK, Johnson M, Morgan J, Miller JN, McMahon WM, Lu J, Jeong EK, Lainhart JE (2007). Diffusion tensor imaging of white matter in the superior temporal gyrus and temporal stem in autism. Neuroscience letters.

[B40] Mottron L, Dawson M, Soulieres I, Hubert B, Burack J (2006). Enhanced perceptual functioning in autism: an update, and eight principles of autistic perception. J Autism Dev Disord.

[B41] Polleux F, Lauder JM (2004). Toward a developmental neurobiology of autism. Mental retardation and developmental disabilities research reviews.

[B42] Herbert MR (2005). Large brains in autism: the challenge of pervasive abnormality. Neuroscientist.

[B43] Casanova MF, van Kooten IA, Switala AE, van Engeland H, Heinsen H, Steinbusch HW, Hof PR, Trippe J, Stone J, Schmitz C (2006). Minicolumnar abnormalities in autism. Acta neuropathologica.

[B44] Mountcastle VB (1997). The columnar organization of the neocortex. Brain.

[B45] Rubenstein JL, Merzenich MM (2003). Model of autism: increased ratio of excitation/inhibition in key neural systems. Genes, brain, and behavior.

[B46] Just MA, Cherkassky VL, Keller TA, Minshew NJ (2004). Cortical activation and synchronization during sentence comprehension in high-functioning autism: evidence of underconnectivity. Brain.

[B47] Horwitz B, Rumsey JM, Grady CL, Rapoport SI (1988). The cerebral metabolic landscape in autism. Intercorrelations of regional glucose utilization. Archives of neurology.

[B48] Uhlhaas PJ, Singer W (2007). What do disturbances in neural synchrony tell us about autism?. Biological psychiatry.

[B49] Brown C, Gruber T, Boucher J, Rippon G, Brock J (2005). Gamma abnormalities during perception of illusory figures in autism. Cortex; a journal devoted to the study of the nervous system and behavior.

[B50] Hughes JR (2007). Autism: the first firm finding = underconnectivity?. Epilepsy Behav.

[B51] Belmonte MK, Yurgelun-Todd DA (2003). Functional anatomy of impaired selective attention and compensatory processing in autism. Brain Res Cogn Brain Res.

[B52] Brock J, Brown CC, Boucher J, Rippon G (2002). The temporal binding deficit hypothesis of autism. Dev Psychopathol.

[B53] Tommerdahl M, Tannan V, Cascio CJ, Baranek GT, Whitsel BL (2007). Vibrotactile adaptation fails to enhance spatial localization in adults with autism. Brain Res.

[B54] Cascio C, McGlone F, Folger S, Tannan V, Baranek G, Pelphrey KA, Essick G (2007). Tactile Perception in Adults with Autism: a Multidimensional Psychophysical Study. Journal of autism and developmental disorders.

[B55] Tommerdahl M, Whitsel BL, Cox EG, Diamond ME, Kelly DG (1987). Analysis of the periodicities in somatosensory cortical activity patterns. Society for Neuroscience Abstracts.

[B56] Tommerdahl M, Favorov O, Whitsel BL, Nakhle B, Gonchar YA (1993). Minicolumnar activation patterns in cat and monkey SI cortex. Cereb Cortex.

[B57] Tommerdahl M, Chiu J, Whitsel BL, Favorov O, Casanova MF (2005). Minicolumnar patterns in the global cortical response to sensory stimulation. Neocortical Modularity and the Cell Minicolumn.

[B58] Chiu JS, Tommerdahl M, Whitsel BL, Favorov OV (2005). Stimulus-dependent spatial patterns of response in SI cortex. BMC Neurosci.

[B59] Favorov OV, Kelly DG (1994). Minicolumnar organization within somatosensory cortical segregates: II. Emergent functional properties. Cereb Cortex.

[B60] Favorov OV, Kelly DG (1994). Minicolumnar organization within somatosensory cortical segregates: I. Development of afferent connections. Cereb Cortex.

[B61] Butterworth S, Francis S, Kelly E, McGlone F, Bowtell R, Sawle GV (2003). Abnormal cortical sensory activation in dystonia: an fMRI study. Mov Disord.

[B62] Nelson AJ, Chen R (2008). Digit Somatotopy within Cortical Areas of the Postcentral Gyrus in Humans. Cereb Cortex.

[B63] Leocani L, Comi G (2006). Movement-related event-related desynchronization in neuropsychiatric disorders. Progress in brain research.

